# Supplementary material for: Deletion of wfs1 Impairs Oligodendrocyte Precursor Cells Dorsal Distribution and Myelination Through the wfs1-hmgcs1 Axis in Zebrafish
Source: Biology (Basel). 2026 Aug 16;15(16):1402. doi: 10.3390/biology15161402 (PMC13510151; doi:10.3390/biology15161402)
Supplement: Supplementary file 1 [file biology-15-01402-s001.zip › File S1-Transcriptome sequencing data and differentially expressed genes (DEGs) in wfs1-- zebrafish at 5 dpf/2.KEGG/wfs1KOvsWT/DOWN/src/dre03018.html]

dre03018


- dre:448949

- Down regulated genes

448949(-1.15683913516418)

- dre:553665

- Down regulated genes

553665(-1.47971352380159)

- dre:566017

- Down regulated genes

566017(-1.85661913518901)

- dre:565659

- Down regulated genes

565659(-1.14717516835564)

- dre:568001

- Down regulated genes

568001\_2(-2.92834331323733)
- dre:561416

- Down regulated genes

561416(-4.80431336765849)

- dre:324574

- Down regulated genes

324574(-1.80226689040574)

Close
